# Whole-Genome Sequencing Characterization of Virulence Profiles of *Listeria monocytogenes* Food and Human Isolates and In Vitro Adhesion/Invasion Assessment

**DOI:** 10.3390/microorganisms10010062

**Published:** 2021-12-28

**Authors:** Giuditta Fiorella Schiavano, Collins Njie Ateba, Annalisa Petruzzelli, Veronica Mele, Giulia Amagliani, Fabrizia Guidi, Mauro De Santi, Francesco Pomilio, Giuliana Blasi, Antonietta Gattuso, Stefania Di Lullo, Elena Rocchegiani, Giorgio Brandi

**Affiliations:** 1 Department of Humanities, University of Urbino Carlo Bo, Via Bramante 17, 61029 Urbino, Italy; 2 Food Security and Safety Niche Area, Department of Microbiology, Faculty of Natural and Agricultural Sciences, Mafikeng Campus, North-West University, Mmabatho 2735, South Africa; collins.ateba@nwu.ac.za; 3 Istituto Zooprofilattico Sperimentale dell’Umbria e delle Marche “Torgo Rosati”, 06126 Perugia, Italy; a.petruzzelli@izsum.it (A.P.); f.guidi@izsum.it (F.G.); g.blasi@izsum.it (G.B.); s.dilullo@izsum.it (S.D.L.); e.rocchegiani@izsum.it (E.R.); 4 Department of Biomolecular Sciences, University of Urbino Carlo Bo, Via Santa Chiara 27, 61029 Urbino, Italy; veronicamele1990@gmail.com (V.M.); giulia.amagliani@uniurb.it (G.A.); mauro.desanti@uniurb.it (M.D.S.); giorgio.brandi@uniurb.it (G.B.); 5 National Reference Laboratory for *Listeria monocytogenes*, Istituto Zooprofilattico Sperimentale dell’Abruzzo e del Molise, 64100 Teramo, Italy; f.pomilio@izs.it; 6 Dipartimento di Sanita’ Pubblica Veterinaria e Sicurezza Alimentare, Istituto Superiore di Sanita’ (ISS), 00161 Roma, Italy; antonietta.gattuso@iss.it

**Keywords:** *Listeria monocytogenes*, Whole-Genome Sequencing (WGS), premature stop codon (PMSC), virulence genes, pork-meat products, adhesion and invasion capacity

## Abstract

*Listeria monocytogenes* (*Lm*) is the causative agent of human listeriosis. *Lm* strains have different virulence potential. For this reason, we preliminarily characterised via Whole-Genome Sequencing (WGS) some *Lm* strains for their key genomic features and virulence-associated determinants, assigning the clonal complex (CC). Moreover, the ability of the same strains to adhere to and invade human colon carcinoma cell line Caco-2, evaluating the possible correspondence with their genetic virulence profile, was also assessed. The clinical strains typed belonged to clonal complex (CC)1, CC31, and CC101 and showed a very low invasiveness. The *Lm* strains isolated from food were assigned to CC1, CC7, CC9, and CC121. All CC1 carried the hypervirulence pathogenicity island LIPI-3 in addition to LIPI-1. Premature stop codons in the *inlA* gene were found only in *Lm* of food origin belonging to CC9 and CC121. The presence of *LIPI2_inlII* was observed in all the CCs except CC1. The CC7 strain, belonging to an epidemic cluster, also carried the internalin genes *inlG* and *inlL* and showed the highest level of invasion. In contrast, the human CC31 strain lacked the *lapB* and *vip* genes and presented the lowest level of invasiveness. In *Lm*, the genetic determinants of hypo- or hypervirulence are not necessarily predictive of a cell adhesion and/or invasion ability in vitro. Moreover, since listeriosis results from the interplay between host and virulence features of the pathogen, even hypovirulent clones are able to cause infection in immunocompromised people.

## 1. Introduction

*Listeria monocytogenes* (*Lm*) is a major foodborne pathogen causing human listeriosis, a severe disease with the highest fatality rates of all other foodborne diseases [1,2]. Invasive infections mainly occur in immunocompromised people, the elderly, pregnant women, and neonates [3,4,5] and are caused by the ability of *Lm* to invade human cells, crossing multiple physiological barriers [6].

Transmission to humans occurs through the consumption of food, mainly ready-to-eat (RTE) foods, including meat and dairy products. Once ingested, *Lm* can invade intestinal epithelial cells, gaining access to the lymphatic system and bloodstream, resulting in the dissemination of the pathogen to the liver, spleen, central nervous system, and, in pregnant women, to the placenta [7].

The ability of *Lm* to adhere to and invade phagocytic and nonphagocytic cells is an important aspect of its pathogenesis, which consist of multiple stages, including cell adhesion, internalisation, vacuolar escape, intracellular replication, movement by actin mobilisation, and cell-to-cell spread [8]. Over the last decade, major advances have been made in understanding the roles of the virulence factors involved in the pathogenesis of *Lm* [9].

The pathogenicity of *Lm* is mediated by a wide range of virulence factors that allow it to infect, survive, and replicate in a variety of host cell types [10]. Thanks to the numerous studies conducted to investigate the adhesion, invasion, and/or virulence regulation of this pathogen, the roles of different virulence factors have been well-characterised in different cell culture or animal models, together with the relative encoding genes [10,11].

In more detail, four Listeria pathogenicity islands (LIPI-1, LIPI-2, LIPI-3, and LIPI-4) have been identified so far [12,13,14,15]. LIPI-1, necessary for intracellular survival and spread, is present in all *Lm* strains and is composed of six genes, including *prfA*, *actA*, *hly*, *mpl*, *iap*, *plcA*, and *plcB*. LIPI-2 is a 22-kb gene cluster involved in phagosome disruption [15]. LIPI-3 is composed by eight genes (*llsAGHXBYDP*) and encodes a biosynthetic cluster involved in the production of Listeriolysin S (LLS), a haemolytic and cytotoxic factor that is known to be required for *Lm* virulence in vivo [14,16]. LIPI-4 is a cluster of six genes and is involved in neural and placental infection [17,18].

Internalin A (InlA) and B (InlB), encoded by the *inlAB* operon, bind the eukaryotic cell membrane receptors E-cadherin and Met and the receptor of the hepatocyte growth factor (HGF), inducing the bacterial uptake through receptor-mediated endocytosis [19]. Many studies have previously reported multiple distinct mutations that lead to premature stop codons (PMSCs) in the *inlA* gene that cause a dysregulated expression of the internalin protein [20,21], significantly decreasing the invasion ability of the mutated strain in human epithelial cells [22].

Other proteins such as fibronectin-binding protein (FbpA), Auto, and Vip are suggested to have a role in mediating *Lm*’s entry into the host cell [8]. In addition, *Lm* utilises the *Listeria* adhesion proteins (Lap A and Lap B) to exploit epithelial defences and cross the intestinal epithelial barriers [8,23].

To date, *Lm* has been classified into four major evolutionary lineages (I, II, III, and IV); 13 agglutination serotypes; five molecular serogroups; and several Multi Locus Sequence Typing (MLST) clonal complexes (CCs) [12,17,24,25].

Serotypes 1/2b and 4b, along with serotype 1/2a, are the main serotypes that cause human disease and represent 90–95% of cases [2,26]. Recent advances in *Lm* infection biology have reported the existence of hypo- and hypervirulent CCs. In particular, certain CCs such as CC1, CC2, CC4, and CC6 are more frequently associated with clinical cases and are hypervirulent in a humanised mouse model, whereas others like CC9 and CC121 are mainly of foodborne origin and show hypovirulence in vivo [17,27].

The methods for determining strain virulence include in vivo bioassays (animal models), in vitro cell assays, and molecular methods to detect virulence genes [28].

Several mammalian cell lines have been used in in vitro studies aimed at evaluating the pathogenic potential of *Listeria* species. Among these, the Caco-2 human colon adenocarcinoma cell line, whose characteristics simulate structural and functional features of mature enterocytes in vitro, has been most widely used to investigate intestinal adherence and invasion, as well as the intracellular replication of *Lm* [29,30].

The analysis of genetic virulence determinants, previously undertaken mainly through PCR detection, is now more frequently performed via Whole-Genome Sequencing (WGS) technology and a bioinformatic analysis with appropriate virulence analytic tools, which are able to detect the genes responsible for the pathogenicity of different strains.

WGS provides the most comprehensive overview of a bacterial strain with the highest possible microbial subtyping resolution compared to the other typing methods. Therefore, WGS has become a new typing standard in public health and food microbiology, replacing the former gold standard typing tools such as PFGE and serotyping. This WGS approach outperforms the traditional methods with respect to robustness, discriminatory power, comparability, ease of data exchange, and cost.

The purposes of this work were to (i) identify via WGS key genomic features and virulence-associated determinants of *Lm* isolates, assigning the clonal complex (CC), and (ii) characterise the ability of the strains to adhere and invade human colon carcinoma cell line Caco-2, evaluating the possible correspondence with their genetic virulence profile.

## 2. Materials and Methods

### 2.1. Bacterial Strains

The strains of *Lm* tested in this study included isolates of food origin (*n* = 6) and strains from human cases of listeriosis (*n* = 3). The characteristics and sources of the *Lm* strains used in this study are reported in Table 1.

The food-derived strains were collected between 2015 and 2016 within the framework of the annual official control plan activity [31,32], carried out in the Marche region for routine and extraordinary control. One of these strains, the 1715, belonged to the same epidemic cluster causing the outbreak.

The human-derived strains were collected during the listeriosis outbreak that occurred in that period, but they did not belong to the same epidemic cluster as the one causing the outbreak. The food matrices included various types of pork meat products, including fresh salami (with seasoning <30 days), salami, spit-roasted pork, and “coppa di testa” (Italian head cheese, a pork-derived meat jelly-seasoned product). This study also included three clinical strains that were isolated between 2013 and 2015 from the blood or cerebrospinal fluid of patients with listeriosis within the Italian surveillance network of human listeriosis coordinated by the Italian National Institute of Health (Istituto Superiore di Sanità, ISS).

The strains were selected in order to test at least one strain for each serotype associated with human disease: 1/2a (human strains 490 and 566; foodborne strains 1608, 2018, and 1715); 1/2b (foodborne strain 1484); and 4b (human strain 1498; foodborne strains 1487 and 1643) [26]. Isolates with identical MLST profiles were identified as distinct strains by WGS. The non-pathogenic *Listeria innocua* (ATCC 33090) was used as a negative control in the adhesion and invasion assays. All *Lm* strains were grown at 37 °C for 16 h in tryptone soya yeast extract broth (TSYEB) and tryptone soya yeast extract agar (TSYEA) (Difco Laboratories, Detroit, MI, USA) supplemented with 0.6% (*wt/vol*) yeast extract (Difco). The medium Agar *Listeria*, according to Ottaviani and Agosti (ALOA), was used for the selective isolation of *Lm*, according to ISO 11290-1-2: 2017.

### 2.2. Whole-Genome Sequencing (WGS)

DNA extraction was performed using a QIAamp DNA Mini Kit (Qiagen, Hilden, Germany) following the manufacturer’s protocols, with minor modifications according to Portmann [33].

The purity of the extracts was evaluated using a NanoDrop 2000 spectrophotometer (ThermoFisher Scientific, Waltham, MA, USA). Starting from 1 ng of input DNA, the Nextera XT DNA chemistry database (Illumina, San Diego, CA, USA) was used for library preparation according to the manufacturer’s protocols. WGS was performed on the NextSeq 500 platform (Illumina, San Diego, CA, USA) with the NextSeq 500/550 mid-output reagent cartridge v.2 (300 cycles, standard 150-bp paired-end reads).

For the analysis of WGS data, an in-house pipeline [34] was used, which included steps for trimming (Trimmomatic v.0.36) [35] and quality control check of the reads (FastQC v.0.11.5). De novo genome assembly of paired-end reads was performed using SPAdes v.3.11.1 [36] with the default parameters for the Illumina platform 2 × 150 chemistry. The genome assembly quality check was performed with QUAST v.4.3 [37].

#### 2.2.1. Multi Locus Sequence Typing (MLST)

The MLST scheme used to characterise *Lm* strains is based on the sequence analysis of the following seven housekeeping genes: *acbZ* (ABC transporter), *bglA* (beta-glucosidase), *cat* (catalase), *dapE* (succinyl diaminopimelate desuccinylase), *dat* (D-amino acid aminotransferase), *ldh* (lactate deshydrogenase), and *lhkA* (histidine kinase) [38] The seven genes of MLST scheme and the clonal complexes (CCs) were deducted in silico using the BIGSdb-*Lm* database (http://bigsdb.pasteur.fr/listeria; accessed on 29 April 2021).

#### 2.2.2. Virulence-Associated Genes Detection

The “*Virulence*” tool of the BIGSdb-*Lm* database (http://bigsdb.pasteur.fr/listeria; accessed on 3 September 2021) was used to detect virulence genes in the genomes of the selected strains. On the basis of the output of the gene presence/absence, a heatmap was generated using Morpheus matrix visualisation and analysis software from the Broad Institute (https://software.broadinstitute.org/morpheus/; accessed on 3 September 2021).

The presence of PMSCs in the *inlA* gene was also investigated. When the BIGSdb-*Lm* database reported that a PMSC mutation was present, the mutation position and the length of the resulting truncated InlA protein were specified [39].

### 2.3. In Vitro Assays

#### 2.3.1. Epithelial Cell Line

Human colon carcinoma epithelial cell line (Caco-2) (ECACC 86010202) cells were obtained from the European Collection of Authenticated Cell Culture (St. Louis, MO, USA).

Caco-2 cells were cultured as monolayers in 75-cm^2^ flasks with Dulbecco’s modified Eagle’s medium (DMEM) containing 10% (*vol/vol*) heat-inactivated foetal bovine serum (FBS), 1% nonessential amino acids, 1% antibiotic solution (100-U/mL penicillin and 100-µg/mL streptomycin), 1% L-glutamine, and 1% sodium pyruvate. Once the flasks reached 90% confluency, the cells were digested using trypsin and seeded at a desirable density onto 6-well plates (Corning Inc., New York, NY, USA).

Plates were incubated at 37 °C and 5% CO_2_ for at least 24 h to achieve full confluency after seeding.

All cell culture materials were purchased from Sigma-Aldrich (St. Louis, MO, USA).

#### 2.3.2. Adhesion Assay

Two days prior to the adhesion assay, Caco-2 cells were seeded in 6-well plates to obtain semiconfluent monolayers (1.5 × 10^5^ cells/mL), as described by Reddy [40], with minor modifications. On the day of the assay, cells were washed with phosphate-buffered saline (PBS, pH 7.4), and fresh prewarmed medium without FBS was added to the wells. Overnight cultures of clinical and food-derived *Lm* strains were grown in TSYEB, with shaking at 200 rpm, and used in the experiment adjusted to an OD_600nm_ = 1.0. Ten-fold dilutions of the cultures were plated onto TSYEA and incubated at 37 °C for 24 h. After incubation, the bacterial concentration of the culture was determined by the number of colony-forming units (CFU).

The Caco-2 cells grown in 6-well tissue culture plates were infected with 10^7^ bacteria to yield a multiplicity of infection (MOI) of approximately 100 CFU per cell. The precise number of inoculated bacterial CFU added at time zero (T_0_) was subsequently calculated according to the plate counts on TSYEA. To synchronise adhesion without forcing adhesion, bacteria were spun down on the cell layer for 1 min at 200× *g*. After incubation at 37° C and 5% CO_2_ for 1 h with bacteria to allow adherence, the monolayers were washed five times thoroughly in cold PBS to remove the bacteria that had not adhered. Serial dilutions were plated on TSYEA and incubated at 37 °C for 24 h; then, the *Lm* colonies were enumerated to determine the number of adhered bacteria.

The adhesion efficiency (%) for each strain was expressed as the percentage of the number of bacteria attached to the cells compared with the total number of CFU provided in the inoculation multiplied by 100. Noninfected wells were used as negative controls, and each assay was performed in triplicate. *L. innocua* ATCC33090 was included as a negative control.

#### 2.3.3. Invasion Assay

Caco-2 cells were infected as described in Section 2.3.2 and incubated at 37 °C and 5% CO_2_. After 3 h post-infection, cells were washed five times with cold PBS, and fresh media containing 50-µg/mL gentamycin (Sigma Aldrich, St. Louis, MO, USA) were added with an additional 90 min of incubation under the same conditions to kill the extracellular bacteria. After incubation, cells were extensively washed with cold PBS to remove gentamycin; then, the intracellular bacteria were recovered by lysis of the monolayers using 500 µL of cold 0.1% Triton X-100 and sonication (Fisher Scientific Sonic Dismembrator Model 100, Pittsburgh, PA, USA, setting 3, 3 pulses, 6 s each).

The resulting suspension was diluted 10-fold, spread on TSYEA, and incubated at 37 °C for 24 h. The number of CFUs was considered as the number of bacteria that had invaded the Caco-2 cells; it was considered that the counts obtained 3 h after the onset of infection represented the number of bacteria that had been internalised. Uninoculated wells were used as negative controls, and each assay was performed in triplicate wells and was repeated at least two times. *L. innocua* ATCC33090 was included as a negative control.

The invasion level (%) of each strain was calculated by dividing the number of CFU that invaded the cells (with gentamycin) by the total number of CFU obtained without gentamycin treatment and was expressed as a percentage.

#### 2.3.4. Hoechst Staining

The adhesion and invasion capacities of *Lm* in Caco-2 cells were qualitatively analysed by fluorescent microscopy. Caco-2 cells were plated and infected as described above (adhesion and invasion assays). Following the process of washing, infected cells were fixed with 4% paraformaldehyde in PBS, permeabilised with cold methanol, and stained using 10 µM with Hoechst 33342 Staining Dye Solution (Sigma-Aldrich, St. Louis, MO, USA). After further washes with PBS, cells were observed under fluorescent microscopy using a “DAPI” filter.

#### 2.3.5. Statistical Analysis

An unpaired, two-tailed *t*-test was applied to evaluate the statistical differences between the adherent bacteria or intracellular bacteria and the reference negative control (*L. innocua* ATCC 33090). Differences were considered significant at *p* < 0.05. The analyses were conducted using GraphPad Prism5 Software.

## 3. Results

### 3.1. Whole-Genome Sequencing (WGS) and Bioinformatics Analysis

Sequences in agreement with the recommended quality control thresholds [41] were obtained for all the strains. The quality metrics for each genome are reported in Table 2.

For each strain, exact matches were found for all the seven genes of the MLST scheme, and the relative clonal complex (CC) was identified.

Three strains belonged to CC1; two belonged to CC121; and the remaining strains belonged to CC7, CC9, CC31, and CC101 (Table 3).

**Figure 1:**
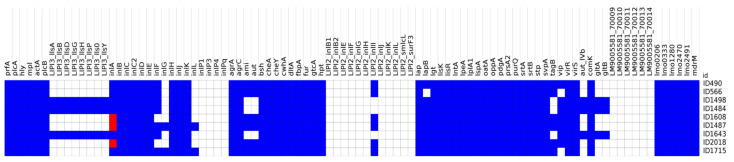
Heatmap of the virulence genes detected in silico using the BIGSdb-*Lm* scheme. Blue: presence of the gene; red: gene with a mutation that leads to premature stop codons (PMSCs); white: absence of the gene.

On a scheme of 92 targets, a total of 71 different virulence genes were detected in the nine analysed isolates. A single isolate possessed between 57 and 66 virulence genes. The presence/absence of virulence genes for each strain are detailed in the heatmap reported in Figure 1.

The results obtained by Amagliani [42] using their rt-PCR targeting *inlC*, *inlJ*, *inlF*, *lapB*, and *lntA* were confirmed except for the absence of *inlF* in *Lm* 1498. In this strain, the WGS analysis identified the *inlF* gene, showing higher sensitivity.

As expected, all the strains carried LIPI-1, which included *prfA*, *actA*, *hly*, *mpl*, *plcA*, *plcB*, and *iap* (recently renamed cwhA), as reported in Figure 1. The CC1 strains also carried LIPI-3 (*llsA*, *llsG*, *llsH*, *llsX*, *llsB*, *llsY*, *llsD*, and *llsP*); the teichoic acid biosynthesis genes *gltA* and *gltB*, and the invasion gene *aut_IVb*. None of the studied *Lm* carried a complete LIPI-2 or LIPI-4 (protein sequences LM9005581_70009–LM9005581_70014). However, the presence of *LIPI2_inlII* (LIV_RS06070) was observed in all the strains except those belonging to CC1. *Lm* 1487 and 1715 also carried the internalin genes *inlG* and *inlL*.

Only human strain 566 lacked the *lapB* and *vip* genes.

*Lm* 1487, 1608, and 2018 showed a PMSC in the *inlA* gene predicting the translation of a truncated InlA protein instead of the full-length InlA of 800 aa. In particular, 1608 and 2018 carried a mutation firstly observed by Olier [43] and described as a PMSC of type 6 by Moura [39]. This mutation is known to produce a truncated form of InlA of 491aa. Strain 1487 instead presented a type 29 PMSC, resulting in a 576-aa length inlA.

All the other strains presented a full-length sequence of the *inlA* gene (Table 3).

### 3.2. Adhesion and Invasion

**Figure 2:**
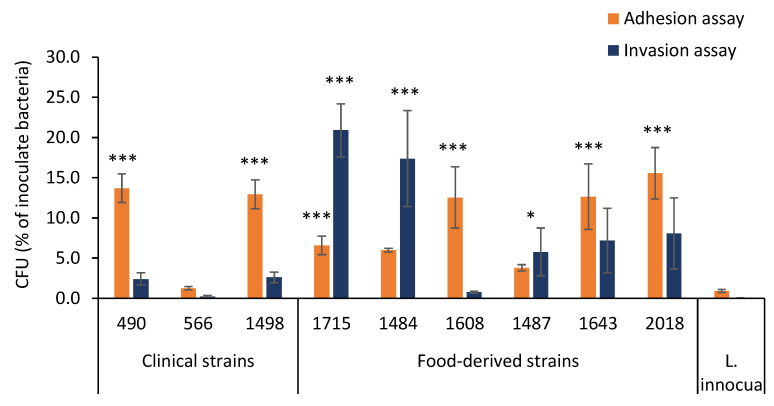
Ability of *Lm* isolates to adhere to and invade Caco-2 human intestinal epithelial cells. Data were plotted as percentages of the starting viable inoculum. Data are the means ± standard deviation (SD) of three separate experiments. *p* < 0.05. The analyses were conducted using GraphPad Prism 5 Software.* *p* < 0.05, *** *p* < 0.001.

All nine *Lm* strains were able to adhere to and invade the Caco-2 cells; the results are detailed in Figure 2.

The levels of adhesion for the clinical strains ranged from 1.25% to 13.70%. Clinical strains 490 and 1498 showed adhesion efficiencies of 13.70% (±3.10%) and 12.94% (±3.11%), respectively, which were significantly (*p* < 0.001) higher than those of *L. innocua*. Conversely, clinical strain 566 showed a lower adhesion efficiency than that of the other strains (1.25%; ±0.35%) and not significantly different to that of *L. innocua*.

The levels of invasion for the clinical strains ranged from 0.24% to 2.61%.

In more detail, strains 490, 1498, and, particularly, 566 showed low invasion levels of 2.40% (±1.69%), 2.61% (±1.47%), and 0.24% (±0.23%), respectively, with no significant difference to the invasion level of *L. innocua*.

The food-derived strains demonstrated a wide variability of adhesion levels, with higher values for strains 2018 (15.55% ± 5.55%), 1643 (12.63% ± 7.08%), and 1608 (12.54% ± 6.57%) and lower values for strains 1715 (6.59% ± 1.99%) and 1484 (6.00% ± 0.40%). Strain 1487 showed the lowest adhesion level (3.78% ± 0.68%).

When compared with *L. innocua* ATCC33090, a high level of significance was found for that of strains 1715, 1608, 1643, and 2018 (*p* < 0.001), whereas, for that of strains 1484 and 1487, no significant differences were found. Adhesion levels were not necessary associated with an increase in the number of bacteria that penetrate the epithelial cells.

The invasiveness of food-derived strains 2018, 1487, and 1643 showed similar percentages (8.06% ± 7.64%, 5.75% ± 5.15%, and 7.19% ± 6.92%, respectively). Strain 1608 showed the lowest invasiveness (0.77% ± 0.19%), while strains 1715 and 1484 presented the highest percentages of invasion (20.90% ± 5.70% and 17.40% ± 1.03%, respectively). Large significant differences (*p* < 0.001) were found between the invasiveness rates of strains 1715 and 1484 and those of *L. innocua.*

**Figure 3:**
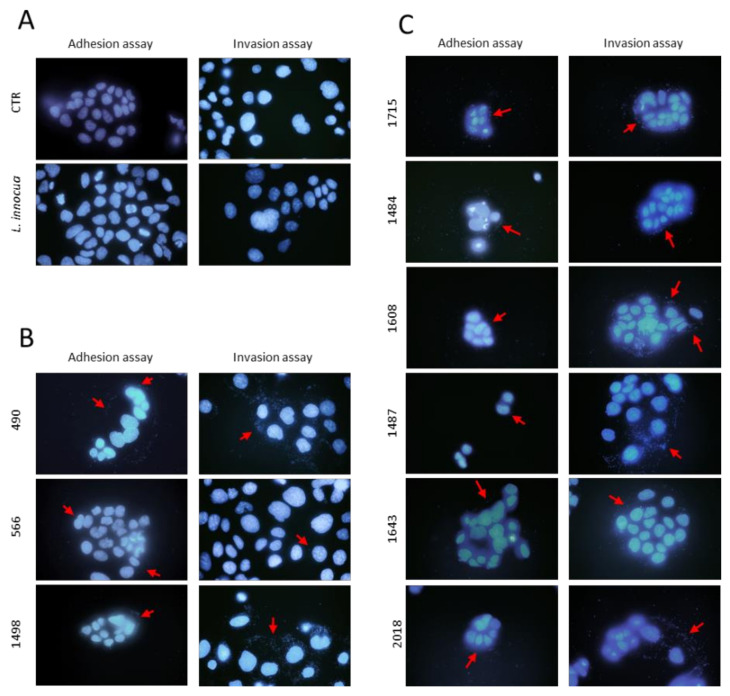
Hoechst staining. (**A**) CTR, uninfected Caco-2 monolayer, and *L. innocua* ATCC33090, negative control. (**B**) Caco-2 monolayer infected with clinical strains. (**C**) Caco-2 monolayer infected with food strains (magnification, 63×). Red arrows highlight Lm infected cells.

The adhesion and invasion of the *Lm* isolates were analysed by fluorescent microscopy staining cells with Hoechst dye (Figure 3).

As expected, neither adhesion or invasion were visible in Caco-2 cells exposed to *L. innocua* ATCC33090 (Panel A)*. Lm* strains of both human and food origin were detectable after the adhesion and invasion assays (Figure 3B,C); however, strain 566 did not show significant adhesive or invasiveness capacity. Due to the difficulty in distinguishing whether the fluorescent bacteria were inside or outside the Caco-2 cells, these data should be interpreted as qualitative.

#### Correlation between Adhesion and Invasion Properties of All Strains

**Figure 4:**
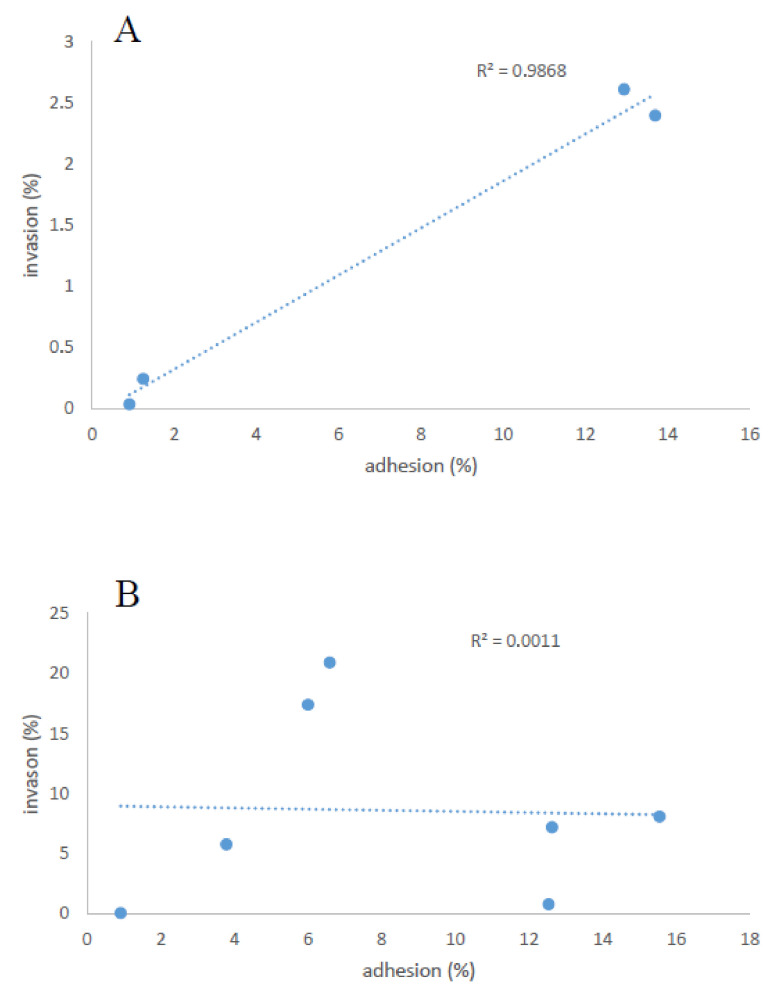
Correlation plot of the adhesion and invasion levels of three clinical (490, 566, and 1498) (**A**) and six food-derived (**B**) (1484, 1487, 1608, 1643, 1715, and 2018) strains of *L. monocytogenes*.

As shown in Figure 4A, in the clinical strains, we found a correlation between the adhesion and invasion levels (R^2^ = 0.9868), whereas no correlation was found between these two indexes in the strains isolated from food (Figure 4B).

## 4. Discussion

*Lm* is an important foodborne pathogen that carries significant public health concerns worldwide. This bacterial species has a great genetic diversity and a wide variability in virulence potential. Several studies focused on *Lm* virulence potential, distinguishing hypo- and hypervirulent clones on the basis of the observed clinical frequency, virulence gene profiles, and in vivo and in vitro assays [17,27,44].

The presence/absence of specific virulence associated determinants is considered a marker of increased or attenuated pathogenicity [10,27]. In particular, virulence factors promoting the adhesion and invasion of phagocytic and nonphagocytic cells, as well as the escaping from the vacuoles, are considered the most relevant in the prediction of virulence potential [12,13,14,15,19]. Previous authors aimed to evaluate the association between the presence/absence of the major virulence determinants and the ability of *Lm* to adhere and invade host cells, obtaining different results [10,13,29].

In this work, we selected nine strains among those previously tested [42] using a rt-PCR method targeting five virulence genes, and we applied WGS to deepen the study of virulence, extending it to a wider panel of genetic markers, evaluating the belonging to hypo- or hypervirulent CCs, and assessing the adhesion and invasion abilities in vitro on the Caco-2 cell lines.

The human strain 566 was assigned to CC31, a clone sporadically isolated from humans and most frequently found in food [39,45]. The belonging of this strain to a clone not defined hypervirulent was consistent with its low invasiveness; its ability to cause disease may have been due to the host’s immunosuppression. The other clinical isolates belonged to CC1 and CC101, previously reported as clinical source-associated CCs, with CC1 being considered one of the most hypervirulent CCs [17,46]. Despite this, these strains showed a low invasiveness during our experiments.

The *Lm* strains isolated from food were assigned to CC1, CC7, CC9, and CC121. As previously reported, CC1 and CC7 are frequently associated with human listeriosis, but they have also been detected in food products [17]. In particular, CC1 is considered the most prevalent clinical CC in several countries, and it is strongly associated with cattle and dairy products [39]. CC7, instead, was previously defined an intermediate MLST clone between those mainly associated with infection and those strongly associated with food and has caused listeriosis outbreaks in the past [47,48]. CC9 and CC121, instead, were previously defined as hypovirulent clones with low clinical frequency that are particularly adapted to food processing environments due to their high resistance to stress [17,27].

Consistent with the above, 1484 and 1715, belonging to CC1 and CC7, respectively, showed the highest level of invasiveness if compared with the other food isolates. The CC1 strain 1498 instead unexpectedly presented a low invasiveness percentage despite having a good level of adhesiveness. In particular, the results obtained for *Lm* 1715 were interesting considering that this strain belonged to the epidemic cluster causing the severe listeriosis outbreak that occurred in Central Italy between 2015 and 2016. *Lm* belonging to CC9 and CC121 showed lower levels of invasiveness.

Investigating the virulence profiles, we observed that the virulence gene count substantially differed only between CC1 strains and all the others. Among the typed strains, in fact, those belonging to CC1 were the only ones carrying LIPI-3 in addition to the widely distributed LIPI-1. Consistently, with these results, LIPI-3 was mainly described in lineage I and was previously reported in CC1 and CC4. It encodes a biosynthetic cluster involved in the production of Listeriolysin S (LLS), a haemolytic and cytotoxic factor conferring a greater virulence to *Lm* [14,17,18]. LLS is expressed only under oxidative stress conditions, and this confers a better ability in terms of phagosome escape. Moreover, pathogenicity studies on murine models demonstrated that LIPI-3 was responsible for the increased virulence of some strains [13].

Among the typed strains presenting LIPI-3, 1484, and 1643 (Figure 1), presented a good level of invasiveness, while 1498 showed an unexpectedly a low level.

PMSCs in the *inlA* gene were found only in *Lm* of food origin, in accordance with Van Stelten et al. 2010, who reported that a significantly greater proportion of RTE food isolates carried such mutations than human clinical isolates, which carried a full-length *inlA*. Moreover, consistent with several studies, all the typed strains presenting a PMSC mutation belonged to CC9 or CC121, and two of them presented a low Caco-2 cell invasion ability in vitro [17,27,44].

The highest percentage of invasiveness showed by strain 1715 could be explained due to the presence in its genome of additional internalin genes (*inlG* and *inlL*). Although the same genes were also carried by *Lm* strain 1487, which did not show the same results in vitro, in this strain, the presence of a PMSC in the *inlA* gene may have reduced the invasion ability.

The teichoic acid biosynthesis genes *gltA* and *gltB* and the invasion gene *aut_IVb*, significantly more frequent among CC1, CC2, and CC6 clones than strains of the other CCs, were consistently detected only in the 1484, 1498, and 1643 strains, all belonging to CC1 [49].

The remaining CC31 (566) and CC101 (490) did not present the particular genetic features of hypo- or hypervirulence previously described. These strains presented low levels of invasiveness, and it was particularly noteworthy for 566. The extremely low level of invasiveness of this strain could be due, at least partly, to the lack of some virulence genes, such as *lapB* and *vip*, or to the observed lower ability to adhere to Caco-2 cells. However, there was not a direct correlation between the level of adhesiveness and the one of invasiveness for all the strains.

Despite the low number of tested strains, we observed the presence of MLST clones having a different virulence potential on a genetic basis, with some of them presenting specific features of hypo- or hypervirulence. However, listeriosis results from the interplay between host and virulence features of the pathogen: the less immunocompromised the host is, the more virulent the *Lm* strain needs to be to cause disease [15]. In this study, we observed the existence of *Lm* clones carrying specific genetic determinants of hypo- or hypervirulence. However, these features were not necessarily predictive of the cell adhesion and/or invasion ability in vitro. The great limitation in performing studies evaluating in vitro virulence on a large number of strains is the use of cell culture models being very laborious and expensive. A future perspective could be to extend the study to a larger number of strains using innovative biological models, such as the use of larvae.

## 5. Conclusions

In this study, we observed that the clinical strains responsible for cases of human listeriosis belonged to both hypo- and hypervirulent CCs and exhibited very low levels of invasiveness, reflecting how the occurrence of the disease may often be favoured by a host’s immunosuppressive state. In contrast, some *Lm* strains isolated from food belonged to hypervirulent CCs and presented good adhesive and invasive properties, highlighting their significant health risk for the consumer. On the other hand, the combined approach of WGS and phenotypic assays can provide new insights, establishing connections with variations in genetic information and phenotypes that influence *Lm* virulence.

## Figures and Tables

**Table 1:** *Listeria monocytogenes* strains typed in this study.

	Strain ID	Source	Serotype
**Human**	490	Blood	1/2a
	566	Blood	1/2a
	1498	Cerebrospinal fluid	4b
**RTE-food**	1484	“Coppa di testa” head cheese	1/2b
	1608	“Coppa di testa” head cheese	1/2a
	1487	Fresh salami	4b
	1643	Salami	4b
	2018	Spit roasted pork	1/2a
	1715	“Coppa di testa” head cheese	1/2a

**Table 2:** Genome assembly quality metrics.

ID	Vertical Coverage	N° Contigs	Total Length (bp)	N50	L50
Lm_490	105.7	46	3,023,546	308,142	3
Lm_566	515.12	59	3,082,646	417,896	3
Lm_1498	219.6	130	2,945,468	556,758	2
Lm_1484	74	50	2,927,103	147,035	1
Lm_1487	104	111	3,079,929	524,763	3
Lm_1608	123	52	3,024,307	563,871	2
Lm_1643	92.4	61	3,023,637	580,655	2
Lm_1715	133	40	2,934,721	437,349	2
Lm_2018	51.1	71	3,123,917	531,830	2

**Table 3:** MLST and *inlA* typing results: clonal complex (CC), *inlA* allele (BIGSdb-Lm), PMSC type, InlA protein sequence type, PMSC position, and predicting InlA length.

**ID Strain**	**Isolation Source**	**CC**	***InlA*** **Allele**	**PMSC**	**PMSC Type**	**InlA Type**	**Mutation Position**	**InlA Length**
490	human	CC101	21	-	-	Full length		800 aa
566	human	CC31	153	-	-	Full length		800 aa
1498	human	CC1	3	-	-	Full length		800 aa
1484	food	CC1	3	-	-	Full length		800 aa
1487	food	CC9	47	+	29	Truncated	1635 (deletion A)	576 aa
1608	food	CC121	49	+	6	Truncated	1474 (C T)	491 aa
1643	food	CC1	3	-	-	Full length		800 aa
1715	food	CC7	2	-	-	Full length		800 aa
2018	food	CC121	49	+	6	Truncated	1474 (C T)	491 aa
